# The Effect of High-Dose Radiation Therapy on Healthy Vertebral Bone Density

**DOI:** 10.7759/cureus.22565

**Published:** 2022-02-24

**Authors:** Ravi Gaddipati, Garrett L Jensen, Gregory Swanson, Kendall Hammonds, Andrew Morrow

**Affiliations:** 1 Radiation Oncology, Baylor Scott & White Health, Temple, USA; 2 Biostatistics, Baylor Scott & White Health, Temple, USA

**Keywords:** bone density, metastasis, radiation therapy, stereotactic body radiotherapy, vertebral compression fracture

## Abstract

Objective

Increased rates of insufficiency fractures are reported after radiation therapy without well-defined causality. Here, we conduct a cross-sectional study on the density change of a non-lesioned vertebral bone after irradiation relative to a control bone in patients with spinal metastases.

Methods

Patients were identified who received radiation therapy for spinal metastases to a region, including an adjacent vertebra without identifiable malignancy on pre-treatment CT. Every patient had an untreated vertebra of a similar type available as a control. A Hounsfield-density calibration curve was used to measure the vertebral body density before and after treatment. Analysis of covariance was used to model vertebral bone density changes with respect to treatment status. Significance was established as *p *< 0.05.

Results

We identified 36 patients who fit the study criteria. The irradiated healthy bone received a median dose of 30 Gy. The median biologically effective dose (BED) was 60 Gy (α/β = 3) and 39 Gy (α/β = 10). Median follow-up imaging intervals between pre-treatment and follow-up CT scans was 13.4 months. Levene’s test was used to confirm the equality of error variance assumption of ANCOVA (p = 0.093). The mean change in the density of the irradiated vertebral bone was -3.59% (95% CI = -8.51% - 1.32%, p = 0.149).

Conclusions

We found no significant change in vertebral bone density attributable to radiation treatment. Further work is needed to elucidate if increased fracture rates after radiation are due to factors other than bone density.

## Introduction

Bone is a frequent site of metastasis, with 70% of cancer patients demonstrating lesions on autopsy [1]. Bone metastases disproportionately affect the spine, leading to chronic back pain, spinal instability, and neurological defects [2]. A clinical benefit of stereotactic body radiotherapy (SBRT) in metastasis has been shown regardless of tumor histology or lesion size. A multi-institutional study of 387 cases found the local control rate was 84% after two years; others demonstrated rates approaching 90% [3-5]. Despite the advantages of local dose control, side effects continue to pose a serious problem. A review of 11 studies covering 2911 spinal segments by Faruqi et al. found that vertebral compression fracture (VCF) is a frequent complication of SBRT, occurring in 5.7%-39% of patients [6].

The pathophysiology underlying the increased VCF rate after radiation is unclear though several studies have demonstrated the deleterious effects of radiation on bone in animal studies. An early study showed late and acute disruptions in blood flow following 0-30 Gy of radiation. Five weeks after treatment, the irradiated bone was unresponsive to angiogenic factors, suggesting a permanent alteration in bone viability [7]. Another study demonstrated decreased alkaline phosphatase activity in mouse tibia following X-rays due to loss of mesenchymal cells [8]. However, clinical data specific to radiation-induced changes in bone is sparse. In 1988, Overgaard followed 231 breast cancer patients treated with mastectomy and chest wall radiation over six years for rib fractures. The fracture rate on follow-up imaging was 48% in the high-dose (BED = 52.0 Gy) radiation group and 6% in the low dose (BED = 41.4 Gy) group [9]. Proposed mechanisms of increased fracture risk include acute inflammation, tumor vascular changes, radiation necrosis, fibrotic remodeling, and increased adiposity [10]. In a single-institution study of VCF, Cunha et al. identified lung or liver metastasis and high radiation dose as independent risk factors for fracture, suggesting that both play a role in the pathogenesis of VCF [11-12].

The objective of this study was to measure the relationship between radiation therapy and spinal bone density while mitigating malignancy effects on density. Previous studies have measured bone density changes in diseased bone following radiation. Palthe et al. measured post-radiation density in sacral chordoma patients and found diseased bone radiodensity, in Hounsfield units (HU), decreased after radiotherapy by 23% after a mean follow-up period of 97 days [13-14]. Interestingly, one study found a 48.4% increase in HU in diseased vertebral bone after radiotherapy [15]. However, many of these studies are limited due to significant disease present in the studied bone. Here, the studied cohort had healthy vertebral bodies included in the treatment field and allowed the measurement of healthy bone density. Vertebrae outside of the radiation field provide same-patient controls for global bone density changes.

## Materials and methods

This study was approved by the Baylor Scott & White institutional review and ethics boards. Patients who were treated with conventional, hypofractionated, or stereotactic radiation for spinal metastasis (Table 1) at our institution were identified between May 2008 and December 2018. Patients included in the study received radiation to a healthy spinal level adjacent to the metastatic level without malignancy. These unaffected vertebrae were included in the treatment field to allow full dose administration to the lesioned bone. Vertebrae 1-3 levels outside of the radiation field were selected as controls. Patients were excluded if a control vertebra was not available, pre or postradiotherapy CT was absent, or new lesions were found on follow-up.

**Table 1:** Demographics

Demographics					
Sex					
Male			20		
Female			16		
Ethnicity					
Caucasian			32		
African American			8		
Other			1		
Primary Malignancy					
Bladder			1		
Breast			8		
Carcinoid			1		
Liver			2		
Lung			5		
Myeloma			4		
Pancreatic			3		
Prostate			12		
Renal			5		
Age					
Median	Min	Max			
69.6	48.0	92.2			
Dose (cGy)					
Median	Min	Max			
3000.0	800.0	4500.0			
Follow-up Period (Months)					
Median	Min	Max			
13.4	2.3	34.6			
Vertebra Type					
Cervical	1				
Thoracic	21				
Lumbar	19				

**Figure 1:**
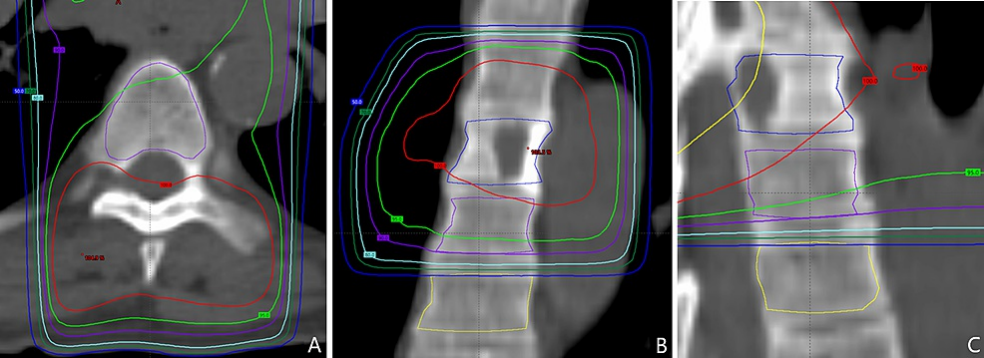
Transverse (A), coronal (B), and sagittal (C) views demonstrating the contouring strategy with isodose lines The lesioned vertebral body is outlined in blue. The density of the adjacent irradiated segment (purple) and control (yellow) was measured in this study.

The density was measured inside regions of interest outlined in Aria (Varian Medical Systems, Richardson, TX) on pre-treatment CT imaging (Figure 1). The trabecular and one-third of the cortical vertebral body were included in the measurement region. One-third of the cortical bone was empirically chosen to avoid biasing the overall density while still capturing cortical density changes. Contours were then transformed onto post-treatment images and alignment verified for follow-up imaging. To obtain the density of the outlined region, each HU measurement was transformed to density with a non-linear calibration curve before averaging values. A CT scanner (GE Optima 580, General Electric Company, Boston, Massachusetts) calibrated to American College of Radiology (ACR) standards was used to image masses of known density encompassing the measured range (CIRS Electron Density Reference Phantom, Model 62, Computerized Imaging Reference Systems Inc., Norfolk, Virginia). The reported density reflects the average of converted Hounsfield units within the outlined region of interest.

An analysis of covariance (ANCOVA) model was used to determine radiation status effects independent of pre-treatment density. Radiation status was defined as the independent variable, post-treatment density as the dependent variable, and pre-treatment density as the covariate. To confirm the assumptions of ANCOVA are valid, Levene’s test was used to verify the equality of group variances and the Shapiro-Wilk test to confirm residual normality. A p-value of 0.05 was established as the threshold for significance.

## Results

Patients (n = 93) were identified who were treated for spinal metastasis at our institution between 2008 and 2018. Of these, 43 patients with a total of 48 treated healthy vertebra had compatible controls outside of the treatment field. Seven patients were excluded due to new lesions in the control vertebra at follow-up. Of the 36 patients included, 20 (56%) were men and 16 (44%) were women (Table 1). The median age is 69.6 (range 48 - 92). There were nine different primary malignancies, with the most common being prostate (n = 12) followed by breast (n = 8). The median total radiation dose was 30.0 Gy (range = 8 - 45 Gy) and the median dose fraction was 3.0 Gy (SD = 0.88 Gy). The median BED was 60 Gy (α/β = 3) and 39 Gy (α/β = 10). Patient follow-up CT scans occurred between 2.3 and 34.6 months with a median time between scans of 13.4 months. Cervical, thoracic, and lumbar levels were represented in the dataset.

**Figure 2:**
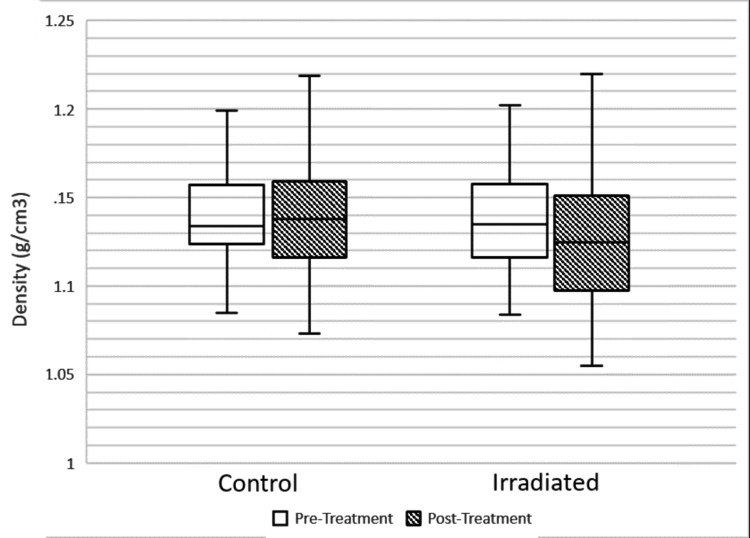
The average density of control and irradiated bone before and after treatment

The average density before treatment of the control group was 1.141 g/cc (SD = 0.033, range = 1.085 - 1.234). The average density of the treatment group before radiation was 1.139 (SD = 0.029, range = 1.084 - 1.202). Following treatment, the average control density was 1.140 g/cc (SD = 0.035, range = 1.073 - 1.237), and the average irradiated density was 1.128 g/cc (SD = 0.045, range = 1.055 - 1.235). The adjusted mean change in the density of irradiated bone after treatment was -3.59% (95% CI: -8.5% - 1.31%, p = 0.18) as shown in Figure 2. Post-treatment density was significantly correlated with pre-treatment density (p < 0.001). The measured densities satisfied the underlying assumptions of the ANCOVA model. Levene’s test confirmed there was no significant difference in variance between the control and treatment groups (p = 0.093). The Shapiro-Wilk test confirmed residual normality (W = 0.925).

## Discussion

In this cross-sectional study, we found there was no significant change in healthy vertebra body density after radiation therapy during a median 13.4-month follow-up period.

Multiple other studies have found changes in bone density after radiation therapy, with varying results. Most similar to this study and also measuring changes in vertebral bone density, Wei et al. demonstrated a dose-dependent decrease in density between scans after completion of radiation therapy and nine to 12 months later. Patients were being treated for abdominal cancers and measurements were not affected by bone lesions as in this study. The change in Hounsfield units ranged from 19.2 Δ%HU (< 5 Gy) to 51.7 Δ%HU (>35 Gy) [16]. There are several limitations of this study that we attempted to address. Rather than using CT-derived HU values, we compared real density. In addition, pre-treatment density was used as the baseline as opposed to post-radiation density. Lastly, Wei et al. measured density as an average of three points in the cancellous bone as opposed to a volumetric average.

Prostate cancer was one of the most common primary malignancies in the studied population. Many of these patients received androgen ablation therapy as part of their treatment. In addition, a portion of the population received bisphosphonate or denosumab therapy between treatment and follow-up. This limitation was mitigated by selecting a nearby control vertebral body. As a result, any systemic changes in bone density were reflected similarly in the control and irradiated bone.

This study has several limitations. This single-institution study was limited to those with pre and post-treatment imaging that encompassed a diseased, a healthy, and a control spinal segment. Density measurement was not possible in those who developed new lesions in the treated healthy or control vertebra during the follow-up period (n = 7). This may have introduced a selection bias, as surgery is often the treatment for compression fractures. We could avoid this, as no VCF was found in the studied healthy bone.

Our results show that there is no significant association of bone density with radiation treatment. This suggests that any increase in bone fragility may be related to other factors. While these results are not generalizable to VCF in diseased bone, they provide perspective on the possible contribution of radiation.

## Conclusions

The pathogenesis of increased compression fractures in patients with spinal metastases who undergo radiotherapy is still unclear. We found no significant change in vertebral bone density attributable to radiation treatment. Further work is needed to elucidate if increased fracture rates after radiation are due to factors other than bone density.
